# The neural thermostat malfunction: revisiting heatstroke through the lens of warm-sensitive neuron dysregulation

**DOI:** 10.3389/fncel.2026.1675202

**Published:** 2026-02-24

**Authors:** Xiaofeng Zhao, Jie Zhu, Qingqiu Tang, He Zhu, Zhifeng Liu

**Affiliations:** 1 The First School of Clinical Medicine, Southern Medical University, Guangzhou, China; 2 Department of Medicine Intensive Care Unit, General Hospital of Southern Theater Command, Guangzhou, China; 3 Department of Pediatric, General Hospital of Southern Theater Command of PLA, Guangzhou, China; 4 Department of Pediatric, Daping Hospital, Army Medical University, Chongqing, China; 5 Department of Clinical Research Institute, Zhanjiang Central Hospital, Guangdong Medical University, Zhanjiang, China

**Keywords:** heatstroke, neural circuit, neuroinflammation, thermoregulation, warm-sensitive neurons

## Abstract

Rising global temperatures have turned heatstroke from a seasonal concern into a year-round public health crisis, yet its underlying neuropathological mechanisms remain elusive. Central to this problem are hypothalamic warm-sensitive neurons (WSNs), the master regulators that integrate central and peripheral thermal signals. This review synthesizes recent advances linking the molecular architecture of WSNs to heatstroke pathophysiology. Emerging evidence redefines WSNs not simply as temperature sensors, but as putative neuromodulatory hubs that may coordinate neuroinflammation, blood-brain barrier (BBB) disruption, and multi-organ failure through hypothesized neurotransmitter-cytokine crosstalk. By mapping the functional hierarchy of WSNs—from molecular thermoregulation to systemic control of thermoeffectors—this work proposes targeted neurotherapeutic strategies, offering a novel neural circuit-based framework for managing heatstroke.

## 1 Introduction

### 1.1 Background

The growing frequency and intensity of heat waves present a mounting global challenge. Beyond direct health risks, they significantly elevate the burden of chronic illnesses and cause substantial economic damage, collectively threatening both physical and mental health. WSNs in the hypothalamic preoptic area are master regulators of body temperature, integrating thermal signals to activate heat-dissipation mechanisms. The central role of thermoregulatory dysfunction in heatstroke is underscored by the vulnerability of these neurons to hyperthermia. Hypothalamic injury, featuring WSNs dysfunction, disrupts autonomic control, creating a pathogenic positive feedback loop that accelerates the transition from manageable heat illness to severe heatstroke with systemic complications. Extreme heat exposure can induce ischemia and oxidative stress in the hypothalamus, compromising WSNs function. This failure creates a vicious cycle: impaired thermoregulation leads to uncontrolled hyperthermia, which further damages WSNs, culminating in the characteristic central nervous system (CNS) dysfunction and multi-organ failure of heatstroke.

In light of the prevailing heat waves and unprecedented temperatures, the prevention and management of heatstroke are imperative. This paper seeks to thoroughly evaluate and analyze the progress on heatstroke and WSNs, with the objective of enhancing the understanding of the pathophysiology of heatstroke through comprehensive discussion. This can establish a theoretical foundation for the advancement of more efficacious therapies for heatstroke.

## 2 Overview of heatstroke

### 2.1 Definition and classification

Heatstroke, a life-threatening and severe condition, is categorized based on pathogenesis and clinical manifestations into classic heatstroke (CHS), primarily resulting from thermoregulatory dysfunction that impairs heat dissipation, and exertional heatstroke (EHS), which is chiefly caused by endogenous heat overproduction (Zhang et al., 2024). CHS frequently affects the elderly and prepubertal children. In the elderly, this is attributed to inadequate physiological regulation of heat stress, chronic illnesses, or an inability to live independently. In prepubertal children, the high body surface area-to-mass ratio leads to increased heat absorption, while their underdeveloped thermoregulatory system, insufficient heat dissipation, relatively low blood volume, and diminished sweating rate further restrict their capacity to manage heat effectively. The disease is more prone to development due to variables such as restricted heat dissipation (Liu et al., 2020). EHS is strongly linked to prolonged high-intensity labor or exercise in hot and humid conditions (Bouchama et al., 2022; Périard et al., 2022), with individual variances and substance addiction potentially heightening vulnerability to elevated temperatures (Reardon and Creado, 2014; Nadesan et al., 2017). Regardless of the variations in etiology, both forms of heatstroke are marked by hyperthermia. When heat accumulation surpasses heat dissipation, core body temperature (CBT) significantly increases, typically exceeding 40.5 °C, with evidence indicating consequent neurological compromise and failure of multiple organ systems as defining features of severe presentations (Leon and Bouchama, 2015; Sorensen et al., 2022).

### 2.2 Pathophysiologic mechanisms

In the pathological progression of heatstroke, the body's thermoregulatory system experiences significant impairment or complete dysfunction, rendering it incapable of successfully mitigating the aberrant elevation in body temperature. This pathological phenomenon is frequently ascribed to the interrelated influences of various factors, such as severe environmental conditions, distinct morphological variations among individuals, and possible clinical risk factors (Cramer et al., 2022). Although a hypothesis suggests that damage to the preoptic area (POA) of the hypothalamus serves as the primary catalyst for the impairment of thermoregulatory function, this theory has yet to be substantiated by adequate scientific evidence (Leon and Bouchama, 2015). Conversely, a hallmark of severe heat stress is its capacity to directly induce cell injury and death, accompanied by pronounced inflammation and coagulation activation. This sequence of pathological alterations encompasses various mechanisms of cell death, including apoptosis, pyroptosis, ferroptosis, necrosis, and PANoptosis, which interrelate and exacerbate one another, forming a vicious cycle that may ultimately result in multi-organ failure and significant impairment of CNS (Iba et al., 2023; Kruijt et al., 2023; Wang et al., 2024).

### 2.3 CNS manifestations

Heatstroke fundamentally results from the disruption of the body's thermoregulatory mechanisms for heat production and dissipation. During decompensation, heat generation surpasses heat dissipation, and cardiac output is inadequate for thermoregulation, leading to a persistent increase in CBT (Iba et al., 2023; Zhang et al., 2024).

**Figure 1:**
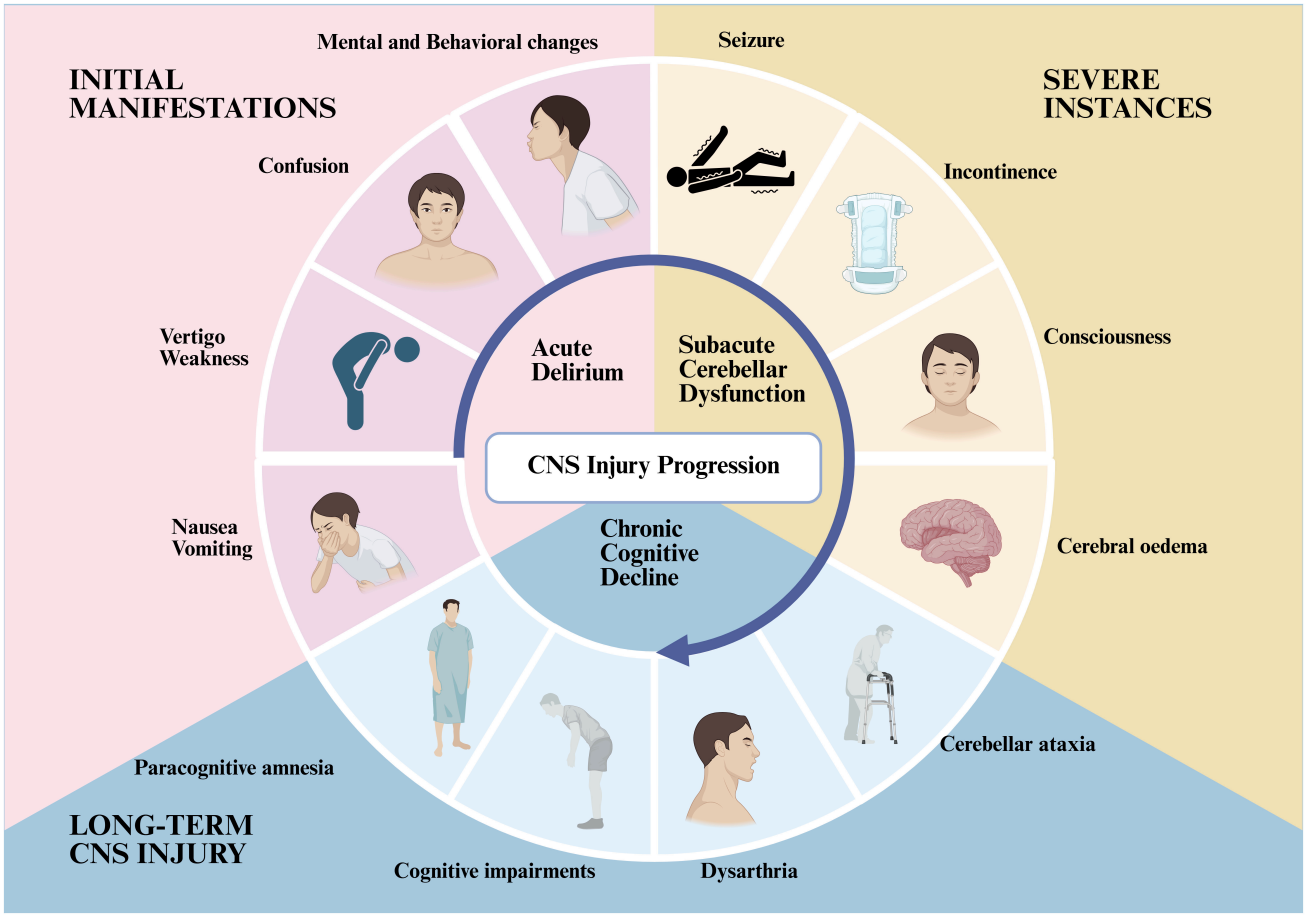
Heatstroke-induced CNS manifestations. This schematic illustrates the temporal progression of CNS injury in heatstroke. It encompasses acute delirium (initial manifestations include confusion, vertigo, weakness, nausea/vomiting, and mental/behavioral changes), subacute cerebellar dysfunction (severe instances include seizures, incontinence, impaired consciousness, and cerebral oedema) and chronic cognitive decline (long-term CNS injury includes paracognitive amnesia, cognitive impairments, dysarthria, and cerebellar ataxia). The directional arrow highlights the sequential development of these phases. Created with BioRender (https://biorender.com).

Initial manifestations of CNS impairment encompass atypical behavior, confusion, vertigo, weakness, emotional instability, aggression, incoherent speech, nausea, and vomiting. In severe instances, seizures and incontinence may arise, frequently accompanied by altered consciousness, which typically resolves when body temperature decreases below 40.5 °C. However, in extreme cases, it may be associated with cerebral oedema (Kruijt et al., 2023; Tan et al., 2024). Certain individuals may endure prolonged CNS injury, including cerebellar ataxia, dysarthria, cognitive impairments, and paracognitive amnesia, potentially persisting for many weeks (Li et al., 2024; Yoneda et al., 2024). Some studies indicate that individuals recovering from heatstroke face an elevated risk of mortality over subsequent months or even years (Shao et al., 2022; Figure 1).

The temporal stages of neuronal injury can be categorized as follows: Acute phase (initial stage of heatstroke onset; Liu et al., 2010; Kourtis et al., 2012): Mitochondrial dysfunction causes heatstroke-induced brain injury. Transcriptomic analysis of the cerebral cortex in heatstroke-affected mice reveals marked acute inflammation. It also reveals activation of mitochondrial apoptosis pathways. There is downregulation of respiratory chain-related genes. And there is disrupted energy metabolism (Li et al., 2020; Fang et al., 2024). Concurrently, heat stress-induced calcium overload further impairs mitochondrial function (White et al., 2007), exacerbating disorders of cellular energy metabolism and apoptosis (Zhu et al., 2024), and potentially triggering PANoptosis (Wang et al., 2024); The subacute phase usually occurs within a few hours to days of the onset of heatstroke. During this phase, heat stress induces persistent neuroinflammation accompanied by the activation of autophagy (Liu et al., 2010; Baindara et al., 2025). Research shows that high temperatures can cause a change in the way synaptic structures in the brain's memory area, called the hippocampus, are arranged, which can affect how they work (Feng et al., 2022). At the same time, increased activity of a protein called caspase-3 can trigger the death of a lot of brain cells (Ji et al., 2019); The chronic phase typically develops over a period of weeks or months following the initial onset. Research has revealed that in mice, following heatstroke, there is a substantial decline in the number of Purkinje cells in the cerebellum, accompanied by demyelination and impaired synaptic function (Miyamoto et al., 2022). Damage to neurons in the hippocampus and alterations in synaptic plasticity may contribute to cognitive impairment (Xie et al., 2024). The function of the thermoregulatory center may be impaired by concurrent neuronal necrosis and neurodegenerative changes (Kourtis et al., 2012).

## 3 Progress in WSNs

In 2016, Zachary A. Knight's team at the University of California, San Francisco, employed advanced technologies such as unbiased RNA sequencing, CRISPR, optogenetics, and specific viral tracers to identify neurons within the Ventromedial Preoptic Area (VMPO) of the hypothalamus. In the VMPO, a subregion of the POA, they identified neurons marked by the expression of both Brain-Derived Neurotrophic Factor (BDNF) and Neuropeptides Pituitary Adenylate Cyclase-Activating Polypeptide (PACAP). Research indicates that these neurons warmth-activated via peripheral thermal signals rather than intrinsically thermosensitive; their response limited to a rather safe, non-destructive temperature range of approximately 31–40 °C (Tan et al., 2016). Upon peripheral injection of capsaicin, an agonist of TRPV1, these neurons marked by the expression of both BDNF and PACAP are capable of receiving and transmitting peripheral temperature information through highly stimulated TRPV1(+) sensory nerve fibers. The activated neurons are crucial for initiating autonomic and behavioral thermoregulatory responses via their distinct projection routes, therefore affecting thermoregulatory systems. In the active state, these neurons induced severe cold-seeking behavior in mice and resulted in a considerable reduction in CBT (Tan et al., 2016). It has been observed that the population of PACAP/BDNF-positive (PACAP/BDNF+) neurons appears to be predominantly not intrinsically thermosensitive. Therefore, the utilization of the term “warmth-activated neurons” (WANs) is preferred for the purpose of differentiating between them and the intrinsically WSNs populations in the POA (Siemens and Kamm, 2018).

To standardize terminology in line with established literature (Tan et al., 2016; Siemens and Kamm, 2018), two key neuronal populations involved in thermoregulation are defined as follows: (1) Warm-sensitive neurons (WSNs): Intrinsically warm-sensitive neurons that detect temperature fluctuations independently without synaptic inputs; (2) Warmth-activated neurons (WANs): PACAP/BDNF+ VMPO neurons primarily driven by peripheral afferent thermal signals and activated by warmth stimulation.

### 3.1 Discovery and localization of WSNs

POA of the hypothalamus has been widely recognized as the region most closely related to thermoregulation (Hammel, 1968). Prior experimental investigations have demonstrated that non-specific activation of the POA can elicit substantial thermoregulatory responses, including panting and sweating, analogous to the behavior of entire organisms subjected to elevated temperatures (Magoun et al., 1938). Conversely, when the POA was compromised, the subjects failed to respond appropriately to decreased body temperature, even in elevated ambient conditions, underscoring the pivotal function of the POA in thermoregulation (Teague and Ranson, 1936; Clark et al., 1939).

The POA has long been considered as a crucial element of the thermoregulatory center based on previous researches. Nevertheless, despite the widespread acknowledgment of this perspective, numerous inquiries remain regarding the specific neuronal types, potential neural circuitry, recognition patterns, regulatory mechanisms, axonal projection pathways, and associated control mechanisms of thermoregulation. The fundamental characteristics of WSNs and molecules remain ambiguous.

The experiments conducted on rats, utilizing acute brain slices, have demonstrated that WSNs manifest an augmentation in their firing rates during periods of elevated temperature (1.07 ± 0.06 impulses s^−1^ °C^−1^). Concurrently, the temperature-induced augmentation in neuronal activity does not engender any modification in their resting membrane potentials (0.06 ± 0.06 mV/°C; Griffin and Boulant, 1995). The investigators determined that ~70% of neuronal units isolated from POA recordings demonstrated autonomous rhythmic discharges independent of external stimulation. It has been determined that between 20% and 40% of all POA neurons demonstrate an elevated rate of firing when subjected to heat, thus classifying them as WSNs (Curras et al., 1991; Griffin et al., 1996; Hori et al., 1999). Recent studies on the thermotolerant plasticity of hypothalamic neurons, when considered in conjunction with the identification criteria proposed in classical literature for thermosensitive neurons, have served to further elucidate the quantitative basis for classification (Boulant and Dean, 1986). Specifically, neurons in which the neuron's temperature coefficient—defined as the change in firing rate per unit temperature change—reaches 0.75–0.8 Hz/°C are classified as thermosensitive neurons (i.e., WSNs); in contrast, neurons exhibiting a temperature coefficient below −0.6 Hz/°C are defined as cold-sensitive neurons (CSNs; Wang et al., 2019; Ambroziak et al., 2025). The previously documented temperature gradient exhibited by the discharge frequency of WSNs (1.07 ± 0.06 impulses/s/°C) has been demonstrated to exceed the established critical threshold for thermosensitive neurons. In addition, it is in alignment with the established classification criteria. This quantitative standard provides a unified reference for accurately identifying WSNs in acute brain slice experiments.

It is important to acknowledge that the proportion of WSNs differs among researches, influenced by the particular hypothalamic region examined, the species investigated, and the criteria employed to assess neuronal thermosensitivity (Boulant and Dean, 1986).

### 3.2 Characteristics and function of WSNs

Thermosensory feedforward signals from the skin are delivered to the POA by afferent pathways through the lateral parabrachial nucleus. In contrast, the central monitoring of CBT is primarily mediated by WSNs in the POA for negative feedback regulations (Nakamura, 2024). The hypothesis posits that the temperature-dependent changes in the firing activity of WSNs may alter the efferent control of thermoregulatory effectors from the POA. This alteration would serve the purpose of correcting deviations in CBT (Nakamura, 2011). The location of projection sites of WSNs remains to be determined. Nevertheless, a substantial body of research has identified a considerable proportion of these neurons as GABAergic (Lundius et al., 2010; Tan et al., 2016).

While the findings of studies about the precise quantity of WSNs that are genuinely inherently temperature-sensitive remain contentious (Hori et al., 1980), the majority of researches indicates that WSNs in the POA can react to temperature fluctuations even without synaptic inputs (Kelso et al., 1982). Irrespective of the specific fraction, these findings strongly indicate that a segment of the WSNs in the POA contains the chemical machinery necessary for detecting temperature variations and transducing them into electrical impulses (Kobayashi et al., 2006).

A series of experiments have demonstrated that optogenetic stimulation of WANs can result in a significant decrease in body temperature. This phenomenon is predominantly attributable to reciprocal fluctuations in thermogenesis and expenditure, accompanied by a substantial augmentation in cold-seeking behaviors (Tan et al., 2016). Consequently, the WANs are crucial to the autonomic and behavioral thermoregulatory processes in animals (Morrison and Nakamura, 2011). These findings contribute to our enhanced comprehension of the thermoregulatory function of distinct warm-responsive neuronal populations (WSNs and WANs) and furnish significant insights for future research endeavors concerning the molecular mechanisms and neural circuits implicated in thermoregulation.

### 3.3 Temperature sensors in WSNs

Temperature-sensitive neurons, a class of nerve cells that respond to temperature changes, are responsible for sensing and regulating temperature homeostasis in the body. Previous studies have investigated temperature sensors to a limited extent, focused on the classic Transient Receptor Potential (TRP) channels and others. TRP belongs to a superfamily of cation-permeable ion channels that function as cellular sensors, integrating diverse environmental stimuli. They are extensively expressed and are crucial in numerous physiological processes (Bais and Greenberg, 2020). The TRPV subfamily comprises six members, which can be classified into two categories: the non-selective cation channels TRPV1-TRPV4 and the more Ca^2+^-selective channels TRPV5 and TRPV6. TRPV1 operates primarily in primary afferent nociceptors, playing a crucial role in the perception of noxious stimuli (e.g., elevated temperatures, capsaicin) and contributing to the mechanism of post-inflammatory thermal sensory hypersensitivity (Rosenberger et al., 2020).

It was hypothesized that TRPV3 and TRPV4 would be significant targets for WSNs, a prediction that was supported by a combination of experimental results and membrane clamp analysis (Kobayashi et al., 2006). It has been noted that other TRP family channels, including TRPV1 and TRPV4, have been referenced in a considerable number of subsequent reports (Güler et al., 2002; Sharif-Naeini et al., 2008; Meng et al., 2012; Sudbury and Bourque, 2013; Zaelzer et al., 2015; Yadav et al., 2017; Li et al., 2025). However, recent findings have revealed the presence of additional molecular markers in WSNs, in addition to the classical TRP channels (Siemens and Kamm, 2018). It is important to note that the VMPO BDNF/PACAP^+^ neurons WANs identified as key warmth-responsive populations have limitations in terms of functional validation. This seminal study relied solely on gain-of-function (GOF) approaches (optogenetic stimulation, capsaicin activation) without complementary loss-of-function (LOF) experiments, and its critical finding regarding inhibitory projections from these neurons has been questioned in subsequent reviews, including by the original authors (Tan et al., 2016; Siemens and Kamm, 2018). A subsequent investigation corroborated that particular and constrained optogenetic stimulation of glutamatergic neurons in the medial preoptic nucleus (MnPO) can replicate the characteristics of leptin receptor (LepR) neurons and culminate in substantial hypothermia (Abbott and Saper, 2017). Within the domain of heat acclimation, the observed warm-sensitive behavior exhibits a pacemaker-like characteristic, driven by a multifaceted integration of increased sodium leak currents and augmented utilization of the NaV1.3 ion channel (Ambroziak et al., 2025). It is important to note that this form of plasticity, which is mediated by NaV1.3 and sodium leak currents and is similar to that seen in pacemakers, may interact with canonical thermosensors (for example, TREK-1/2, which are directly gated by temperature) and NALCN (a key regulator of neuronal resting excitability; Kang et al., 2005; Yang et al., 2022). Together, these provide alternative but complementary mechanisms for thermally induced neuronal excitability modulation during the process of heat acclimation.

Further research has indicated that the transient receptor potential ion channel melastatin-2 (TRPM2) is involved in the POA's response to heat stress when temperatures reach 38 °C or higher. However, this role is not without unresolved literature controversies: Song et al. (2016) initially proposed TRPM2 as a hypothalamic warm-sensitive thermoreceptor (Song et al., 2016), but Kamm et al. (2021) challenged this conclusion with phenotypic data showing that TRPM2 deficiency does not abolish the warm sensitivity of WSNs (Kamm et al., 2021). Instead, the current body of evidence converges on TRPM2's function in mediating thermally induced synaptic outputs/plasticity and sustained hypothermia—rather than serving as an intrinsic membrane heat-gating channel in native POA neurons, as the latter function lacks direct biophysical validation (Kamm et al., 2021). This finding highlights the critical function of TRPM2 as a warm-sensitive thermoreceptor located in the POA. In addition, chemogenetic activation and inhibition of hypothalamic TRPM2-expressing neurons *in vivo* resulted in a decrease and increase in body temperature, respectively. However, subsequent research has established that TRPM2 deficiency, while impacting overall thermoregulation to some degree, does not result in a total loss of thermoregulatory function (Song et al., 2016). In a separate report, further results have been demonstrated that demonstrate that the deletion of the TRPM2 gene does not result in the abolition of the warm sensitivity of WSNs. Recent findings have revealed that the channel exerts a thermal influence on synaptic outputs, which regulate the activity of WSNs. This finding supports the hypothesis that temperature-sensitive synaptic input from the POA local network may serve as a regulatory mechanism, monitoring the intrinsic (temperature-sensitive) activity of POA neurons, thereby contributing to a more intricate thermoregulatory control system. It was observed that thermode-driven, warming-induced, long-lasting hypothermia was dependent on TRPM2, which could be explained by the ion channel's capacity to mediate thermally induced synaptic plasticity. Consequently, the hypothesis was proposed that the capacity to adapt to persistent thermal challenges may be an additional function of synaptic temperature detection and integration (Kamm et al., 2021). These findings offer a novel insight into the precise mechanism of TRPM2's involvement in thermoregulation.

**Figure 2:**
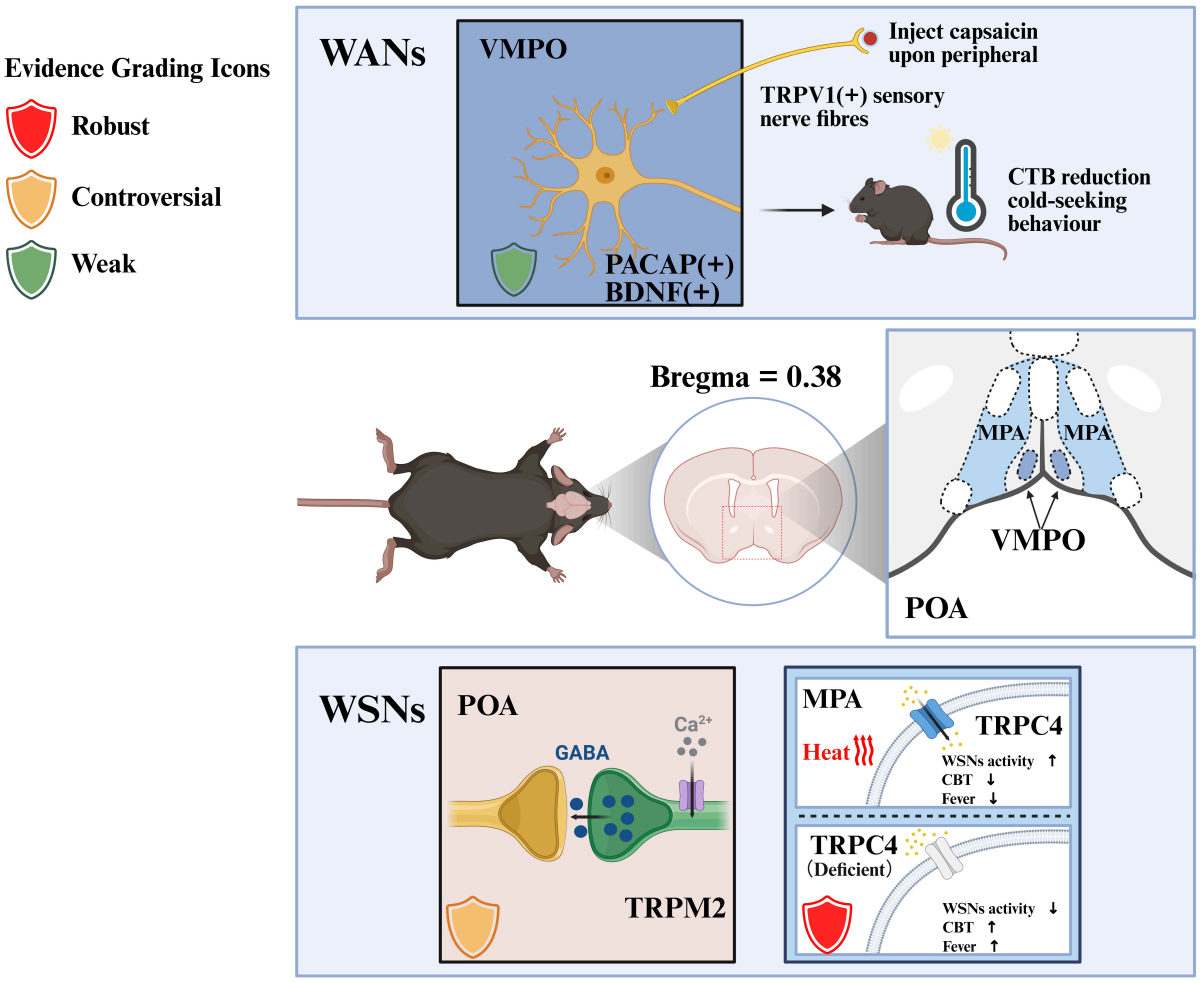
Temperature sensors in WSNs. This figure illustrates the molecular and neuronal mechanisms of WSNs in thermoregulation across distinct POA subregions. Evidence grading icons: Red shield = Strong evidence (multimodal validation with GOF/LOF experiments, no major literature controversies); Green shield = Weak evidence (GOF-only validation or unresolved controversies); Orange shield = Controversial evidence (conflicting literature with unproven core function). Annotated elements: POA subregions (VMPO, ventromedial preoptic area; MPA, medial preoptic area). Note on controversial molecules: TRPM2 was initially proposed as a hypothalamic warm-sensitive thermoreceptor (Song et al., 2016), but subsequent studies challenged this conclusion with weak phenotypic data, supporting its role in thermally induced synaptic outputs/plasticity rather than intrinsic warm-sensing of WSNs (Kamm et al., 2021). In contrast, TRPC4 is validated as a core sensor for internal warmth detection and core body temperature set-point maintenance (Zhou et al., 2023), with consistent GOF/LOF evidence. Created with BioRender (https://biorender.com).

In a separate study, researchers investigated the mapping of thermosensitive nuclei throughout different subregions of the POA. Light-stimulation heating was administered to the MnPO, VMPO, Medial Preoptic Area (MPA), Ventral Part of the Lateral Preoptic Nucleus (vLPO) and the Lateral Preoptic Nucleus (LPO) of the hypothalamus. Heating the MPA and MnPO produced the most pronounced hypothermic effect. To elucidate the precise mechanism underlying this hypothermic effect, the researchers inhibited glutamatergic or GABAergic neurons in the central region of the preoptic area (cPOA; Bregma = 0.0–0.38 mm), focusing on the main body that encompasses the MPA instead of targeting BDNF neurons. The experimental results indicated that this move nearly eradicated the hypothermic effect caused by MPA heating (Zhou et al., 2023). In a further investigation, the researchers discover that the knockdown or silencing of the TRPC subfamily Transient Receptor Potential Canonical-4 (TRPC4) in the GABAergic WSNs within the MPA lead to an elevated basal core temperature, compromised thermal defenses, and heightened feverish symptoms. This discovery indicates that TRPC4, functioning as a core cytoreceptor for the MPA-localized GABAergic WSNs, plays a pivotal role in mediating internal warmth detection and maintaining the core body temperature set-point—a fundamental aspect of WSNs physiology (Zhou et al., 2023). Furthermore, TRPC4 is indispensable for regulating CBT to maintain the set-point temperature, hence preventing hyperthermia and constraining fever. Notably, its functional role is supported by both gain-of-function and loss-of-function experiments with consistent results, with no major contradictory literature (Zhou et al., 2023). Nonetheless, despite these advancements, the processes pertaining to the function of excitatory glutamatergic neurons in the WSNs and their principal molecular targets remain unidentified (Zhou et al., 2023; Figure 2; Table 1).

**Table 1:** Functional properties of thermoregulatory neuronal populations in the POA.

**Location/ marker**	**Cell markers have been identified**	**Thermoregulation-related functional properties**	**Model system**	**Manipulation**	**Evidence strength**	**Notes**
MnPO LepRb^+^	Leptin receptor	• Pharmacogenetic activation promotes heat dissipation and induces hypothermia; • Heat acclimation enhances its warm-sensitive activity to improve heat tolerance.	Rodent (mouse)	Optogenetic stimulation, pharmacogenetic activation, viral tracing, heat acclimation training.	Moderate-strong (*in vivo* functional validation with multiple techniques).	Meets all inclusion criteria; key warmth-activated neuron population. Note: Functional validation relies solely on GOF approaches (no LOF experiments), and the inhibitory projection findings in the original study have been questioned in subsequent reviews (including by the same authors).
VMPO BDNF/PACAP^+^	Brain-derived neurotrophicfactor, Pituitary adenylatecyclase-activatingpolypeptide	• Selectively activated by environmental warmth; • Optogenetic excitation triggers cold-seeking behavior and CBT reduction; • Critical for heat acclimation via postsynaptic potentiation.	Rodent (mouse)	Capsaicin injection, optogenetic stimulation, specific viral tracing, RNAscope.	Moderate-strong (multimodal validation: behavioral, thermal, and neural tracing data).	Meets all inclusion criteria; key warmth-activated neuron population. Validation relies solely on GOF experiments (optogenetics, capsaicin activation) without LOF verification (Siemens and Kamm, 2018).
POA TRPM2^+^	Transient receptor potential melastatin 2	• Mediates POA response to warm stimuli (≥38 °C); • Regulates neural progenitor cell survival during hyperthermia; • Enhances WSN activity via inhibitory synaptic modulation.	Rodent (mouse)	Chemogenetic activation/inhibition, thermode-driven warming, gene knockout, 2-APB administration.	Moderate (*in vivo* functional data; unresolved literature controversies between foundational and subsequent studies; no biophysical proof of intrinsic membrane heat-gating in native POA neurons).	Meets all inclusion criteria. Note: Key controversies exist in literature: Song et al. (2016) proposed TRPM2 as a hypothalamic heat sensor, but Kamm et al. (2021) challenged this with weak phenotypic data, supporting TRPM2's role in thermally induced synaptic outputs/plasticity rather than intrinsic warm-sensing of WSNs.
MPA TRPC4^+^	Transient receptor potential canonical 4	• Essential for internal warm sensing in GABAergic WSNs; • Prevents CBT exceeding set-point via intrinsic thermosensitivity; • Modulates fever responses.	Rodent (mouse)	Gene knockdown/silencing, light-stimulation heating, glutamatergic/GABAergic neuron inhibition, electrophysiological recording.	Strong (multimodal validation: behavioral + molecular + electrophysiological).	Meets all inclusion criteria; pivotal for internal warmth detection and core body temperature set-point maintenance (a fundamental aspect of WSN physiology). Validated by GOF/LOF experiments at genetic (knockdown), molecular (electrophysiology), and pharmacological levels; multimodal verification including behavioral (body temperature/activity), molecular, and electrophysiological analyses with consistent results.
MPO PGD2^+^	Prostaglandin D2 synthase	• Classified as warm-sensitive neurons (dF/dT > +0.8 Hz/°C) via electrophysiological recording; • Temperature elevation (37–40 °C) increases firing rate to promote PGD2 production; • PGD2 activates DP1 receptors in vMPO to induce hypothermia.	Rodent (mouse)	Chemogenetic activation (hM3Dq), patch-clamp recording, TTX/DP1 antagonist (MK-0524) administration.	Moderate-strong (multimodal validation: electrophysiology + pharmacology; 85% overlap with warm-sensitive neurons).	Meets Table 1 inclusion criteria: MPO localization, Ptgds molecular marker, direct warm-sensing validation (dF/dT > +0.8 Hz/°C). PTGDS expression in the POA is relatively broad, even less specific than that of TRPM2, which should be considered when evaluating its specificity as a WSNs marker.

MnPO, medial preoptic nucleus; VMPO, ventromedial preoptic area; MPA, medial preoptic area; POA, preoptic area; CBT, core body temperature; WSNs, warm-sensitive neurons; HS, heatstroke; PTGDS, Prostaglandin D2 synthase; DP1, PGD2 receptor.

**Inclusion criteria:** (1) Localized in the POA or its key subregions (MnPO/VMPO/MPA); (2) Equipped with distinct, identifiable molecular markers (receptors/ion channels/neuropeptides/enzymes) for specific target; (3) Directly validated to participate in physiological thermoregulation (warm sensing or heat-defense effector regulation) via *in vivo*/*in vitro* functional studies (electrophysiology/optogenetics/chemogenetics).

**Evidence strength definition:** Strong: Multimodal validation (behavioral + molecular + electrophysiological) with gain-of-function (GOF) and loss-of-function (LOF) experiments at genetic, molecular, and pharmacological levels; consistent results across studies, and no major contradictory evidence; Moderate-strong: Multimodal validation (behavioral + molecular + electrophysiological) but solely based on GOF experiments (no LOF validation); consistent results but lack of cross-species replication; Moderate: Functional *in vivo* evidence with single-technique validation or unresolved literature controversies, limited biophysical data.

**Note on Heatstroke (HS) Relevance:** All entries are based on physiological thermoregulation evidence; their potential roles in HS (e.g., mediating temperature dysregulation) are inferred from functional properties, as direct HS model validation is currently lacking.

TRP channels are expressed in a variety of tissues and organs, including sensory neurons, skin, and abundantly expressed in the brain (Ramsey et al., 2006). The following structures have been identified as containing TRPM2: the hippocampus, cortex, and dorsal root ganglion sensory neurons of the spinal cord (Ko et al., 2019, 2025). Furthermore, TRPC4 is expressed predominantly in the olfactory bulb, amygdala and hippocampus (Zechel et al., 2007; Koivisto et al., 2022). Despite the absence of specific quantitative data, extant mouse whole-brain single-cell spatial transcriptomic atlas indicate that the expression levels of TRPM2 and TRPC4 in the POA are relatively low (Han et al., 2025). However, according to the latest research, these molecules TRPM2 and TRPC4 contribute to thermoregulation by modulating thermally induced synaptic outputs (TRPM2) and warmth detection/set-point control in GABAergic WSNs (TRPC4); direct intrinsic heatgating of native POA neurons by these channels has not been biophysically confirmed at the mechanistic level, rather than modulating synaptic thermosensitivity.

In light of the distinct characteristics exhibited by the aforementioned regions, it is conceivable that these molecules may also play a significant role in associated vital physiological processes. In the event that TRPM2/TRPC4 are WSNs sensors, it is imperative to ascertain the mechanisms through which local molecules can be specifically regulated, or how the effects on other regions can be avoided during widespread brain stimulation. Furthermore, it is imperative to ascertain whether TRPM2 and TRPC4 are indeed heat-sensitive channels. In contrast to the peripheral TRPM8 and TRPV1 channels, which function as thermosensitive sensors located peripherally, there is an absence of compelling biophysical data substantiating the thermosensitive nature of TRPM2 and TRPC4 channels. A further question that arises pertains to the necessity of two channels (TRPM2 and TRPC4) for central thermosensitivity. Concurrently, the question arises as to whether TRPV1 is the sole factor responsible for temperatures exceeding the threshold in peripheral regions. These questions require further research to clarify them.

In addition to the TRP family channels, prostaglandin D2 synthase (PTGDs)-positive neurons in the medial preoptic area (MPO) represent another well-characterized thermosensitive neuronal population (Wang et al., 2019). These neurons exhibit intrinsic warm sensitivity (temperature coefficient: dF/dT > +0.8 Hz/°C), with temperature elevation (37–40 °C) promoting PTGDS-mediated PGD2 secretion. Released PGD2 then binds to DP1 receptors on VMPO neurons, initiating a hypothermic response characterized by enhanced heat dissipation and reduced thermogenesis (Wang et al., 2019). Functional validation via chemogenetic activation, patch-clamp recording, and pharmacology (DP1 antagonist MK-0524) has confirmed their pivotal role in physiological thermoregulation (included in Table 1; Wang et al., 2019). Notably, PTGDS expression in the POA is relatively broad, even less specific than that of TRPM2, which should be considered when evaluating its specificity as a WSNs marker.

It is noteworthy that estrogen receptor alpha (ERα)-expressing POA neurons (encoded by the ESR1 gene) were excluded from Table 1 despite their temperature-related functions. This is due to the fact that their thermosensitivity is restricted to postpartum female-specific thermal preference remodeling (which is tied to maternal reproductive adaptation) rather than universal warm sensing/heat defense (Zhang et al., 2020). Experiments demonstrated that these neurons mediate postpartum females' reduced thermal preference via altered warm/cold activation, changes absent in non-postpartum or male individuals. As they lack validation in general thermoregulatory contexts, they fail to meet the inclusion criteria for universally relevant WSNs populations as outlined in Table 1.

Paying attention to species differences is vital. While most current animal studies focus on rodents (Song et al., 2016; Tan et al., 2016; Zhou et al., 2023), their thermoregulatory mechanisms differ from those of humans and their WSNs density and responsiveness vary across species (El Bitar and Le Bars, 2015; škop et al., 2020; Tavares and Freire, 2021). Ethical and technical constraints prevent optogenetic/chemogenetic research in humans, which poses challenges for clinical translation.

## 4 Systemic integration of WSNs dysfunction

It is hypothesized that neuronal dysfunction triggered by heat stress (particularly cyclic heat waves) is not an isolated event of central involvement. Indeed, studies have demonstrated that transcriptomics in aged mice under heat stress reveals that heat stress simultaneously triggers intestinal barrier disruption (Xiao et al., 2015; Du et al., 2023), hepatic metabolism disruption (Roy et al., 2024), and BBB damage. Consequently (Chao et al., 2015; Guo et al., 2024), this may results in central neuronal dysfunction through the formation of a putative inflammatory cascade via the gut-hepatic-brain axis (Shapiro et al., 1986; Lin et al., 2009; Xiao et al., 2015; Luo et al., 2023). This finding indicates that the dysfunction of WSNs under heatstroke conditions cannot be interpreted separately from the systemic stress network, and that all of the above mechanisms may directly or indirectly regulate the excitability and survival of WSNs in the POA (Zhang Z. et al., 2021; Du et al., 2024).

### 4.1 Gut-brain axis: enteric-derived endotoxin triggers a neuroinflammatory cascade that impairs neuronal excitability in the preoptic area

In the context of heatstroke, prolonged exposure to elevated temperatures has been demonstrated to trigger oxidative stress pathways within intestinal epithelial cells (e.g., Nuclear factor erythroid 2-related factor 2/Heme oxygenase-1 imbalance), resulting in a substantial diminution of tight junction protein expression (e.g., occludin, Zonula Occludens-1) and consequent impairment of barrier function (Xiao et al., 2015; Du et al., 2023). Concurrently, the process of apoptosis in intestinal epithelial cells is initiated, leading to the exacerbation of barrier damage (Liu et al., 2017). Collectively, these mechanisms enhance intestinal permeability, thereby facilitating the entry of endotoxins (e.g., lipopolysaccharide, LPS) into the bloodstream from the gut, thus inducing systemic inflammatory responses (Shapiro et al., 1986; Lin et al., 2009; Lim, 2018; Li et al., 2020; Luo et al., 2023; Baindara et al., 2025). Furthermore, the condition of heatstroke has been demonstrated to induce an increase in the proportion of gut microbiota such as Bacteroidetes/Firmicutes, whose reduced production of short-chain fatty acids (SCFAs) has been shown to further exacerbate systemic inflammation (Huang et al., 2023).

During this process, inflammatory cytokines such as interleukin-1β (IL-1β), interleukin-6 (IL-6), and tumor necrosis factor-α (TNF-α) have been shown to be elevated in both experimental animals and heatstroke patients (Hammami et al., 1997; Helwig and Leon, 2011; Geng et al., 2015). In addition, in a study of mice subjected to stimulated with LPS and heatstroke, it was observed that NOD-like receptor family, pyrin domain-containing 3 (NLRP3) inflammasome-mediated IL-1β expression was detected in the POA (Zhang Z. et al., 2021). Subsequent studies further confirmed that heat stress induces excessive Reactive Oxygen Species (ROS) production in microglia, which in turn activates the NLRP3 inflammasome in both *in vivo* mouse models and *in vitro* cultured BV2 cells. This process has been shown to induce a cascade amplification effect involving ROS, NLRP3, and proinflammatory factors, thereby further promoting the aforementioned pathological processes (Du et al., 2024).

It is proposed that the aforementioned mechanisms provide a foundation for the following hypothesis: During the occurrence of heatstroke, the presence of enteric endotoxins [resulting from intestinal barrier disruption (Shapiro et al., 1986; Lin et al., 2009; Xiao et al., 2015; Luo et al., 2023)] may trigger a systemic-central inflammatory cascade via the gut-brain axis (Lim, 2018) The overproduction of ROS by microglia is hypothesized to be a pivotal mediator in this process, inducing an increase in NLRP3 inflammasome-mediated IL-1β elevation within the POA (Zhang Z. et al., 2021; Du et al., 2024). Although direct evidence is limited, IL-1β may bind to TLR4/IL-1R receptors on WSNs-a notion that remains to be tested-thereby potentially inhibiting the opening efficiency of voltage-gated ion channels on neuronal membranes and reducing action potential firing frequency, with mechanistic analogs supported by prior studies (Helwig and Leon, 2011; Geng et al., 2015). Moreover, the presence of excess ROS has been demonstrated to oxidatively damage the function of WSNs mitochondrial complexes, thereby reducing ATP generation and consequently inhibiting sodium-potassium pump (Na+/K+-ATPase) activity. This results in neuronal resting potential depolarization and elevated excitability thresholds. With regard to the aforementioned pathway—“enteric endotoxin → ROS → NLRP3 → impaired excitability of WSNs”—there is an absence of definitive systematic reports at present. Nevertheless, we hereby present a key mechanism hypothesis that awaits validation.

### 4.2 Abnormal hypothalamic signaling due to liver dysfunction

The liver, the core metabolic organ, is responsible for maintaining biological metabolism and physiological balance (Watt et al., 2019). Heat stress has been demonstrated to induce multidimensional damage to the liver. Specifically, acute heat stress has been shown to induce metabolic disorders through oxidative stress (Lin et al., 2006), while chronic heat stress has been demonstrated to induce hepatocyte apoptosis via endoplasmic reticulum (ER) stress, accompanied by impaired ammonia detoxification, elevated blood ammonia levels, worsening liver pathology, and impaired hepatic autophagy (Ma et al., 2022; Tang et al., 2022).

The liver-brain axis, a pivotal communication network integrating the gut-liver-brain, has been implicated in the etiology of metabolic and immune-related diseases (Yan M. et al., 2023). In the context of heat stress, multi-organ transcriptomic analysis has been shown to identify shared stress-related genes and pathways between the liver and brain. This analysis has revealed enhanced activity in specific pathways, including chemokines, neuroinflammation, and adipokines. Key molecules, such as the hepatocyte-derived cytokine Orosomucoid 2 (ORM2), were also identified in this study (Roy et al., 2024). As indicated by earlier research, ORM2 has been found to be linked to metabolic-associated steatohepatitis (MASLD) and has a role in the regulation of hepatic and systemic lipid homeostasis (Li et al., 2023). It has been demonstrated that ORM2 secretion increases significantly during periods of heat stress in response to intestinal inflammation and dysbiosis. This finding positions ORM2 as a potential novel biomarker for liver disease (Roy et al., 2024).

In consideration of preceding reports on OR2's function in neuroinflammation and ischemic stroke occurrences, in conjunction with its correlation with astrocyte-derived ORM2 (Jo et al., 2017; Wan et al., 2019), subsequent studies have demonstrated heightened ORM2 levels in the hippocampal and frontal cortical regions of the brain during periods of heat stress, without concomitant elevations in ORM2 mRNA within the brain. This finding indicates that ORM2 is not secreted by astrocytes themselves, but rather, it is more probable that ORM2 is secreted by hepatic hepatocytes and may enter the central nervous system via a compromised BBB (Roy et al., 2024). Concurrently, systemic inflammation induced by heat stress, for instance elevated levels of IL-6 (Castell et al., 1989; Hammami et al., 1997; Ridker and Rane, 2021), interacts with neuroinflammation mediated by central ORM2 (Jo et al., 2017; Wan et al., 2019). This interaction may disrupt the hypothalamic signaling regulation of WSNs in the POA region, resulting in abnormal excitability and dysfunction, a mechanism that requires further experimental validation.

### 4.3 BBB damage: exacerbation of hypothalamic oxidative injury

Heatstroke has been demonstrated to disrupt the BBB and exacerbate oxidative damage in the hypothalamus. In a rat model of heatstroke, increased glial fibrillary acidic protein (GFAP) expression in the hypothalamic region and Evans Blue leakage, indicating BBB impairment, have been observed. Concurrently, oxidative stress markers such as 2,3-Dihydroxybenzoic Acid (2,3-DHBA) and nitrogen oxides (NOx–), along with proinflammatory cytokines like TNF-α and IL-1β, were elevated, accompanied by neuronal apoptosis and degeneration (Chao et al., 2015). A further study reported findings of swelling, vacuolation, and nuclear condensation in neurons and glial cells within the hypothalamic tissue of heatstroke rat models. Observations of neuronal and glial cell swelling, vacuolation, and nuclear condensation were made in hypothalamic tissue. A significant decrease in neuron counts was observed at 7 days post-injury, with some neurons disintegrating or even disappearing. Levels of inflammatory factors increased markedly, while levels of glutathione decreased significantly (Guo et al., 2024). In summary, BBB disruption exacerbates hypothalamic oxidative damage by increasing the influx of peripheral injury factors and impairing local antioxidant capacity (Chao et al., 2015; Guo et al., 2024). It is hypothesized that this may represent a critical pathway affecting the function of WSNs under heatstroke conditions, an idea that awaits further verification (Table 2).

**Table 2:** Evidence map of the gut-liver-brain axis mediating WSNs excitability dysfunction in heatstroke.

**Pathway step**	**Evidence level**	**Primary references**	**Species/ model**	**Notes**
Heat stress → Intestinal barrier disruption → Endotoxemia	Direct	Li et al., 2020; Tavares and Freire, 2021; El Bitar and Le Bars, 2015; Chao et al., 2015; Shapiro et al., 1986; Du et al., 2024	Rat, mouse	Multi-species validation; directly detects increased intestinal permeability and endotoxin translocation; (Li et al., 2020) focuses on heat stress-induced intestinal injury
Heat stress → Elevation of systemic cytokines (IL-1β, IL-6, TNF-α)	Direct	Lim, 2018; Huang et al., 2023; Helwig and Leon, 2011	Rat, Human (heatstroke patients)	Dual support from clinical samples and animal experiments; directly measures elevated levels of systemic proinflammatory cytokines
Heat stress → Central (POA) microglial ROS/NLRP3 activation → IL-1β elevation	Direct	Luo et al., 2023; Lin et al., 2009	Mouse (*in vivo*), BV2 cells (*in vitro*)	Validated by both *in vivo* and *in vitro* experiments; directly detects ROS production, NLRP3 activation, and IL-1β upregulation in the POA
Heat stress → BBB disruption → Hepatic ORM2 entry into the central nervous system	Indirect	Xiao et al., 2015; Du et al., 2023; Roy et al., 2024; Yan M. et al., 2023; Li et al., 2023	Mouse, rat	BBB disruption is direct evidence; ORM2 entry into the CNS is indirectly inferred from increased central ORM2 levels and absence of cerebral synthesis
Central IL-1β/ORM2 → Abnormal WSN channel function/excitability	Speculative	Lim, 2018; Huang et al., 2023 (mechanistic analogs)	–	No direct experiments on WSNs; supported by mechanistic analogy of “cytokines inhibiting neuronal excitability” from general neuronal studies

This table systematically summarizes the key steps of the gut-liver-brain axis pathway involved in WSN excitability impairment during heatstroke, with stratification by evidence strength, primary supporting references, and experimental species/models.

**Column definitions:** (1) “Pathway Step” lists the sequential mechanistic links from heat stress to WSNs dysfunction; (2) “Evidence Level” classifies evidence as “Direct” (experimental direct validation), “Indirect” (inferred from related mechanisms), or “Speculative” (logical deduction without direct experimental support); (3) “Primary References” corresponds to the manuscript's citation numbers for traceability; (4) “Species/Model” indicates the experimental subjects used to generate the evidence; (5) “Notes” briefly clarifies the characteristics of the evidence (e.g., multi-species validation, *in vivo*/*in vitro* experiments). This map enhances the transparency of the evidence chain and facilitates the evaluation of each mechanistic link's credibility.

## 5 Correlation between heatstroke and WSNs

The correlation between heatstroke and WSNs has aroused interest in physiological and pathological research, and a thorough examination of the response mechanisms of WSNs is crucial for comprehending the pathophysiology and progression of heatstroke (Bouchama et al., 2022).

### 5.1 Response patterns of WSNs

Research indicates that the response patterns of WSNs demonstrate considerable variety, with sensitivity to temperature fluctuations differing based on neuron type and anatomical location (Xiao and Xu, 2021). The specifics are as follows:

#### 5.1.1 Alternation of transitory inhibition and excitation

A minor variation of 1 °C in local temperature can swiftly elicit a response in WSNs, characterized by persistent transient inhibition or excitement. This reaction is marked by a sudden alteration in neuronal firing frequency at the onset of temperature change (Kim et al., 2022).

#### 5.1.2 The consistency and variety of response

Various types of WSNs exhibit both uniformity and notable disparities in their reactions to thermal stimuli. The responses of certain neurons to variations in brain temperature are disrupted when the ambient temperature lowers, whereas they stay comparatively constant when the ambient temperature increases (Hellon, 1972). This indicates that WSNs may assume varying regulatory functions in distinct physiological conditions.

#### 5.1.3 The regulatory function of chemical interventions

Excessive chemical interventions influencing the open and closed states of particular ion channels can proficiently govern the functionality of WSNs, hence modulating their response to thermal stimuli and significantly impacting the thermoregulatory mechanism. For instance, 2-aminoethoxydiphenylborate (2-APB), a powerful inhibitor of TRPM2, TRPM3, and stromal interaction molecule 1 (STIM1)/Orai1 ion channels, and an activator of TRPV1, TRPV2, and TRPV3 ion channels (Hu et al., 2004; Xu et al., 2005; Togashi et al., 2008), can reversibly disrupt the temperature response of WSNs in the POA (Song et al., 2016).

### 5.2 The effect of heatstroke on WSNs

Heatstroke is a severe condition resulting from extended exposure to elevated temperatures and humidity, with its onset intricately linked to the functional condition of WSNs.

#### 5.2.1 The impact of elevated temperatures on WSNs

Research indicates that heatstroke induces aberrant neuronal activity, resulting in disrupted motor cortex function and compromised executive function, potentially leading to physical and cognitive deficits (Cramer et al., 2022; Tan et al., 2024). Despite the absence of direct evidence, reports have indicated that elevated temperatures can induce alterations in carbon dioxide and pH levels, resulting in acid-base imbalances that adversely impact the functionality of WSNs (Dean, 2007; Wright and Boulant, 2007). This phenomenon is closely associated with the established pH/CO_2_ sensitivity of WSNs. Research has demonstrated that hypothalamic WSNs exhibit a high degree of responsiveness to extracellular acidosis and elevated CO_2_ levels, with acidosis exerting a direct inhibitory effect on WSNs firing activity (Wright and Boulant, 2007). This malfunction may perpetuate an elevation in body temperature and ultimately precipitate heatstroke. Furthermore, the adaptive response of WSNs in elevated temperature environments may display distinct properties, such as modifying firing patterns in response to prolonged high-temperature stimuli (Tan et al., 2016). Nonetheless, this adaptive reaction may be ineffective at exceedingly high temperatures. This is due to the fact that severe acidosis associated with extreme hyperthermia can override such compensatory plasticity and sustain WSNs dysfunction.

#### 5.2.2 Neuron death and brain injury

While widely acknowledged as the thermoregulatory center of the body, the hypothalamus is nevertheless vulnerable to the effects of heat stress. The damaging effects of heatstroke on nerve cells have been well-documented (Liu et al., 2009), and the phenomenon of inflammation as a result of the condition has also been demonstrated. Another report utilized immunohistochemistry to demonstrate that iNOS accumulates in the hypothalamus in rat heatstroke models (Hsiao et al., 2007). Subsequent immunohistochemistry revealed substantial elevations in GFAP, ionized calcium binding adapter molecule 1 (Iba1), nuclear factor-kappa B (NF-κB), and Cyclooxygenase-2 (COX-2; Chauhan et al., 2017). A recent study has indicated that the levels of myeloperoxidase, TNF-α, and interleukin-1 (IL-1) are increased in the hypothalamus of rats exposed to heatstroke (Tai et al., 2021). These factors may also be significant in explaining the dysfunctionality of WSNs during the development of heatstroke.

#### 5.2.3 Short-term and long-term effects

The principal cause of this is heatstroke (Yoneda et al., 2024), which has resulted in significant damage to the cerebellum, manifesting in ataxia, balance disorders and dizziness (Van Stavern et al., 2000; Jung et al., 2017). Damage to the cerebral cortex has been demonstrated to result in cognitive impairment, impaired consciousness and memory deficit (Fang et al., 2024). Similarly, damage to the hippocampus has been shown to cause memory deficit (Chauhan et al., 2021). At present, there is a paucity of relevant research to further explore the short-term and long-term effects of heatstroke on WSNs.

#### 5.2.4 Possible biological mechanisms

Intracellular calcium excess and endoplasmic reticulum stress response generated by elevated temperatures may constitute a biological mechanism contributing to WSNs injury (Li et al., 2024). These physiological processes can disrupt normal cell function, resulting in neuron death and further neurological impairment.

### 5.3 The role of WSNs in the prevention and management of heatstroke

**Figure 3:**
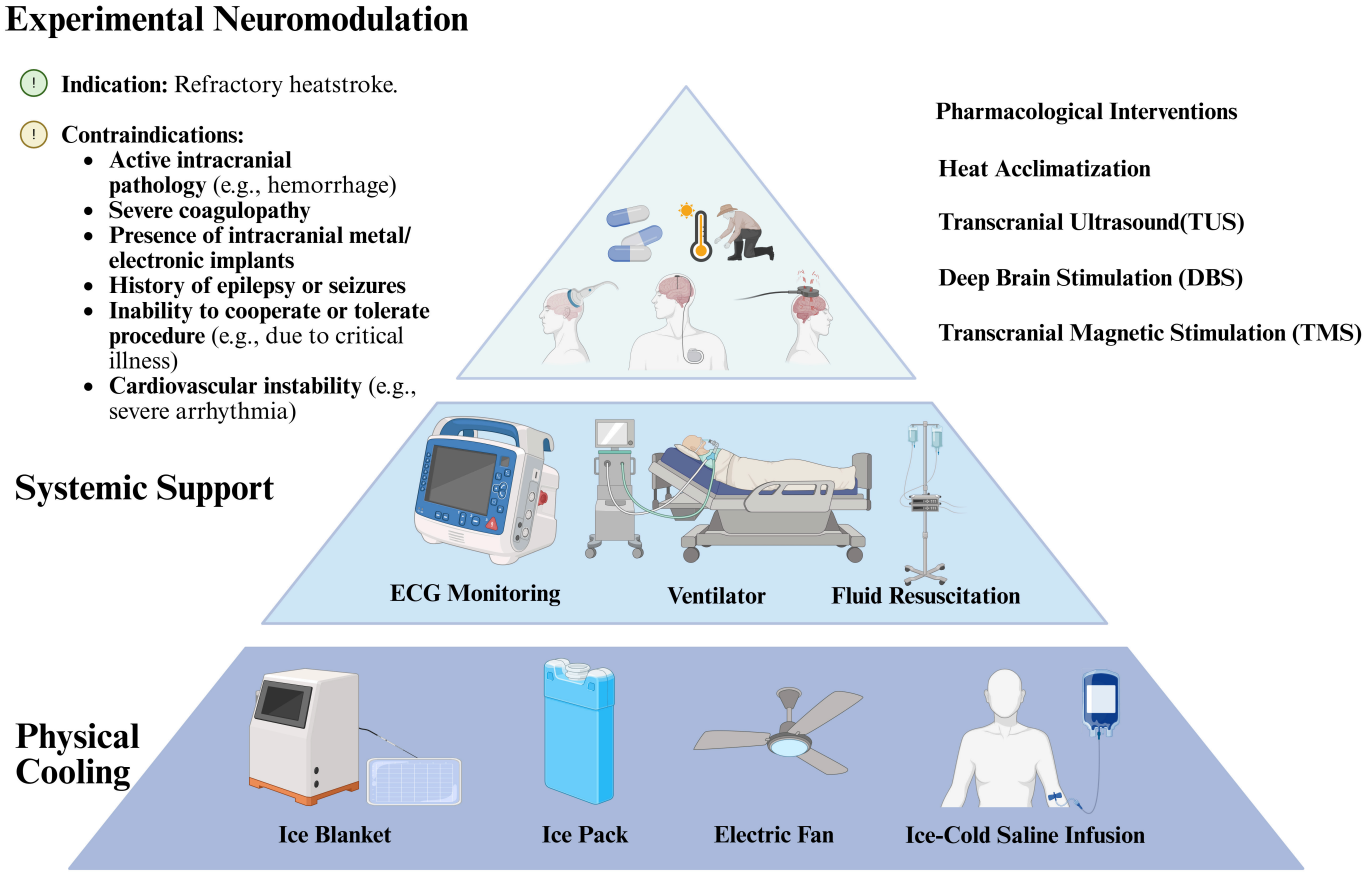
Clinical decision pyramid for heatstroke management. This figure illustrates a tiered framework for making clinical decisions, structured as a pyramid. It guides the intervention for heatstroke from the most basic level of physical cooling (e.g., an ice blanket or an infusion of ice-cold saline) through to intermediate levels of systemic support (e.g., ECG monitoring or fluid resuscitation) and finally to the most advanced level of experimental neuromodulation (e.g., transcranial ultrasound or deep brain stimulation). Created with BioRender (https://biorender.com/).

In the clinical management of heatstroke, a thermoregulatory condition, there is an urgent demand for more controlled molecular targets to enhance therapy techniques (Nakamura and Morrison, 2011; Hemmelgarn and Gannon, 2013; Cheshire, 2016). Several studies have shown that hypothermic neuroprotection is effective (Drury et al., 2010). Nonetheless, aside from antipyretic medications for fever management, there is a scarcity of pharmaceuticals that directly influence thermoregulation, making physical cooling methods the predominant approach at present (Galinsky et al., 2017; Song et al., 2025). However, issues remain, such as insufficient cooling speed for severe cases of heatstroke, a short duration of effect and the possibility of masking the severity of the condition (Bouchama et al., 2022; Chen L. et al., 2025). Due to their distinctive biological features and functions, WSNs present various novel potential approaches for the prevention and treatment of heatstroke (Figure 3).

#### 5.3.1 Potential for pharmacological interventions

Targeting WSNs may lead to the development of novel medications aimed at improving their functional responsiveness in extreme temperature settings. TRPM2 and TRPC4 are currently recognized as potential key molecular targets for WSNs (Song et al., 2016; Zhou et al., 2023), and have already produced results in other fields.

The transmembrane protein TRPM2, an established redox sensor, has been identified as a potential target for hypoxic brain injury. This protein is involved in neuronal death via the generation of reactive ROS (Zhan et al., 2016; Sakaguchi and Mori, 2020). Its antagonists have been demonstrated to protect cells from ROS-induced death (Fourgeaud et al., 2019). In mouse models of cardiac arrest and cardiopulmonary resuscitation, TRPM2 inhibition has been shown to enhance functional recovery after cerebral ischemia (Dietz et al., 2020). In consideration of the fact that TRPM2 is present in high concentrations within the hippocampus of patients diagnosed with major depressive disorder, it can be posited that the channel might serve as a viable candidate for therapeutic intervention in depression (Ko et al., 2019). Furthermore, it has been identified as a susceptibility gene for bipolar disorder (Xu et al., 2006). Furthermore, TRPM2–/– mice have been observed to manifest social cognitive impairments, and some bipolar disorder patients carry TRPM2 gene variants (Xu et al., 2006), thereby further confirming the association between TRPM2 and bipolar disorder (Jang et al., 2015).

The hypothesis that centrally acting TRPC4 antagonists have the capacity to alleviate visceral and neuropathic pain, or at the very least render it more tolerable, has been postulated (Miller et al., 2011; Wei et al., 2015). A series of behavioral studies was conducted on TRPC4–/– mice, yielding positive results in models predicting anxiolytic and antidepressant action (Riccio et al., 2014). This effect could be replicated by a TRPC1/TRPC4/TRPC5 pan-inhibitor, HC-070, which implies a therapeutic potential to treat anxiety disorders (Just et al., 2018). In addition to this, the TRPC4–/– rats exhibited a reduced propensity for self-administered cocaine, in the absence of any concomitant deficits in the acquisition of natural rewards (Rasmus et al., 2013). In the event that this observation is corroborated in human subjects, a TRPC4 antagonist could potentially exhibit clinical efficacy in the treatment of addiction (Koivisto et al., 2022).

Therefore, the development of targeted drugs could offer a new approach to controlling body temperature in the clinical treatment of heatstroke. However, there are still some difficulties to overcome, such as how to specifically regulate the targets of WSNs in particular brain regions.

#### 5.3.2 Transcranial ultrasound (TUS)

As an advanced technology in neuromodulation that uses low-intensity ultrasound waves to target deep brain areas and accurately control neural activity non-surgically (Mohammadjavadi et al., 2022; Sarica et al., 2022). In contrast to conventional non-invasive stimulation techniques like Transcranial Direct Current Stimulation (tDCS), TUS can profoundly stimulate the brain's deeper regions and offer a more extensive therapeutic effect (Zhang T. et al., 2021). The efficacy of TUS in alleviating depressive symptoms, mitigating anxiety, and facilitating cerebral recovery post-stroke (Servick, 2020; Chen et al., 2022; Sarica et al., 2022). Although there are few reports on the application of TUS in heatstroke, it has been studied that TUS can be used to stimulate the thermoregulatory central POA (Pang et al., 2021). Ultrasound deep brain stimulation (UDBS) is another ultrasound technique that can stimulate specific structures or regions in the deep brain. Ultrasound stimulation of freely moving mouse POA through UDBS has been shown to significantly reduce body temperature in mice, a finding that provides support for the potential of ultrasound stimulation as a method of temperature regulation (Pang et al., 2022). In another report, TUS stimulation of POA neurons in rodents, with the dorso-medial hypothalamus as a downstream brain region, followed by inhibition of thermogenic brown adipose tissue, resulted in non-invasive, precise, and safe induction of numb-like hypothermia and hypometabolism (Yang et al., 2023). Subsequent single-nucleus RNA sequencing of POA neurons has identified TRPM2 as an ultrasound-sensitive ion channel. The suppression of this channel has been demonstrated to attenuate ultrasound-induced hypothermia and hypometabolism (UIH; Yang et al., 2023). A separate report delineated a sonogenetic approach that has the capacity to selectively manipulate designated cells, thereby activating specific neural pathways and inducing particular behavioral responses. Sonogenetic stimulation of the subthalamic nuclei of mice with a Parkinson's disease model has been demonstrated to enhance motor coordination and mobility (Xian et al., 2023). Transcranial focused ultrasound (tFUS) stands out as a specialized type of ultrasound technology. It employs specially crafted transducers to produce high-frequency ultrasound waves capable of penetrating the skull and converging on specific brain regions for highly precise neuromodulation. Researchers have discovered that excitatory and inhibitory neurons exhibit intrinsic differences in their responses to the ultrasound pulse repetition frequency (PRF), enabling tFUS to non-invasively target specific neuron types by adjusting the PRF (Yu et al., 2021). Furthermore, recent advancements have demonstrated the potential for establishing a connection between ultrasound and neurons at the genetic level. This development signifies a significant step forward, paving the way for novel applications in biomolecular imaging and sonogenetic control (Rabut et al., 2020).

#### 5.3.3 Transcranial magnetic stimulation (TMS)

TMS is a non-invasive brain stimulation method that utilizes electromagnetic induction, applying a changeable magnetic field through the scalp to generate an electric current that influences neuronal activity in the brain. Repetitive TMS (rTMS) is extensively utilized in the treatment of psychiatric illnesses, including depression and anxiety, while exhibiting fewer adverse effects (Lefaucheur et al., 2020; Eldaief et al., 2022). According to the literature, TMS has been demonstrated to be efficacious in the modulation of body temperature in subjects with obesity. The treatment has been shown to reverse obesity-induced alterations in heat production and dissipation, and this effect appears to be mediated by a mechanism involving the sympathetic nervous system (SNS; Ferrulli et al., 2022). BDNF occupies a pivotal role in neuroplasticity and the functionality of neurons that are sensitive to temperature. Research endeavors have indicated that TMS has the potential to influence the concentrations of BDNF in both serum and cerebrospinal fluid, thereby exerting an indirect modulation on the activity of temperature-sensitive neurons (Chervyakov et al., 2015). A recent report has indicated the development of a magnetogenetic system, comprising a fusion protein composed of an anti-ferritin nanobody and a TRPV1 receptor. This system has been observed to regulate neuronal activity in response to exposure to magnetic fields. In Pitx2-Cre Parkinsonian mice, this resulted in reduced c-Fos expression and motor rotational behavior. The findings indicate that magnetogenetic constructs possess the capacity to modulate the activity of specific neuronal circuits within the intact organism, in a bidirectional manner, and in a manner that is not invasive. This modulation can be achieved through the utilization of clinically available devices (Unda et al., 2024). This finding further reinforces the enormous potential for regulating body temperature by modulating specific neural circuits in the future.

#### 5.3.4 Deep brain stimulation (DBS)

DBS regulates neuron activity by delivering electrical currents to specific areas of the brain. It has made significant progress in recent years in several areas, such as movement disorders, depression, and anxiety disorders (Figee et al., 2022; Potel et al., 2022; Guo et al., 2023). Research indicates that DBS aimed at the hypothalamus can influence temperature perception, elevate cold sensation thresholds and cold pain thresholds, and enhance the body's ability to detect and react to temperature variations more effectively in hot environments (Jürgens et al., 2009; Galhardoni et al., 2019). According to the literature, DBS targeting the lateral hypothalamic area has been shown to induce frequency-dependent modulation of both sleepiness level and CBT (Davin et al., 2024). DBS of the hypothalamic paraventricular nucleus (PVN) has been reported to activate the metabolism of Brown adipose tissue (BAT) through a mechanism that may include the activation of local GABAergic receptors, thereby affecting body temperature levels (Mota et al., 2023). Moreover, research conducted on mice has demonstrated that DBS of the WSNs in the medial preoptic nucleus (MPN) can result in the induction of tolerable hypothermia (Zhang et al., 2022). This direct finding provides further evidence supporting the future application of DBS to stimulate the WSNs of heatstroke patients, thereby controlling body temperature.

#### 5.3.5 Heat acclimatization (HA)

Individuals engaged in athletic activities and those employed in elevated temperature settings face a significant risk of heatstroke. For example, good heat acclimatization can only be achieved after working in a hot environment for 100 minutes a day for 7–14 days. Heat acclimatization is manifested by increased cardiac output and sweating, less sodium in sweat than usual, and twice the normal heat dissipated through sweating (Périard et al., 2021). A comprehensive understanding of the role of WSNs could enhance the formulation of preventive strategies for these groups. For instance, augmenting the functionality of WSNs via acclimatization training enhances individual heat tolerance (Ambroziak et al., 2025; Chen B. et al., 2025; Li et al., 2025). In a recent report, a distinct group of neurons in the POA of the mouse hypothalamus was identified that increases its activity rheostatically over the course of heat acclimation, thereby providing a foundation for further investigation into the neural mechanisms underpinning thermoregulation. This property is critical for the development of heat tolerance in mice. This pacemaker-like, warm-sensitive activity is driven by a combination of an increased sodium leak current and an enhanced utilization of the NaV1.3 ion channel. This salient neuronal plasticity mechanism adaptively drives acclimation, thereby promoting heat tolerance (Ambroziak et al., 2025). Another report indicated that HA has the capacity to potentiate preoptic TRPV1 neurons, thereby contributing actively to the defense against heat via the medial Preoptic Area (mPOA)-Dorsomedial Hypothalamic Nucleus (DMH)/Raphe Pallidus Nucleus (RPa) circuit during EHS. This results in the suppression of adipose tissue thermogenesis and the facilitation of vasodilation. They found that delivering exosomes engineered with RVG-Lamp2b-Irisin significantly improves the function of mPOA TRPV1 neurons, providing a promising preventive strategy for EHS (Li et al., 2025). Similarly, a further study revealed that heat acclimation in mice necessitates preoptic BDNF neurons and postsynaptic potentiation (Chen B. et al., 2025). These researchers provide new evidence regarding the relationship between WSNs and HA, and highlight the importance of integrating WSNs monitoring to establish enhanced scientific training regimens that can effectively mitigate the occurrence of heatstroke.

In conclusion, WSNs possess significant potential in the prevention and treatment of heatstroke, and their distinctive biological characteristics and roles facilitate the development of innovative therapeutic approaches. Recent advancements in brain stimulation techniques have yielded notable outcomes in the management of conditions such as epilepsy, anxiety, depression, and stroke. However, there are still many challenges to overcome in translating these findings from the laboratory to clinical application. The main focus is on the following areas: First, technical barriers, most current research has focused on rodents rather than non-human primates. Although both are warm-blooded, their temperature regulation mechanisms differ. The path from laboratory research to clinical practice is long and arduous. Furthermore, the penetration effectiveness of non-invasive methods (such as TUS and TMS) through the skull must be considered. The introduction of novel materials as adjuncts to prefrontal localization and targeting accuracy gives rise to new challenges in the clinical translation process (Unda et al., 2024). Furthermore, the potential risk of intracranial heating during implementation requires thorough discussion. Secondly, the safety and feasibility of certain procedures and the ability of critically ill patients to cooperate with treatment determine whether such technologies can be implemented. The question of whether invasive implants, such as DBS, can be tolerated by some critically ill patients remains unresolved. Conversely, non-invasive approaches (e.g., TUS and TMS) necessitate patient cooperation, sedation, imaging examinations, or preoperative localization. It is imperative to acknowledge that these procedures are associated with inherent safety risks, which include the potential triggering of epilepsy, arrhythmias, and the exacerbation of oxidative damage. These issues require further discussion in conjunction with clinical realities. In conclusion, it is imperative to address the ethical and practical challenges associated with the implementation of these technologies. The integration of these innovations into clinical practice is contingent upon the availability of specialized equipment and trained personnel. Additionally, there has been a steady progression in the field of thermoregulation, though the application of these techniques in treating elevated body temperature remains limited. Therefore, it is imperative to investigate the potential of related methodologies to enhance patient care. In the future, comprehensive research and technology advancements are anticipated to yield significant breakthroughs in the treatment of heatstroke.

## 6 Conclusion and future perspectives

Heatstroke, a significant thermoregulatory disorder, primarily depends on physical cooling methods in clinical settings. However, certain patients continue to experience elevated temperatures and unfavorable outcomes post-treatment. Consequently, the investigation of novel molecular targets for the regulation of body temperature has emerged as a critical focus in clinical research. WSNs are integral to thermoregulation, and their precise manipulation is anticipated to serve as a novel approach to heatstroke treatment.

Molecular targets like TRPC4 and TRPM2 play significant roles in thermoregulation. TRPC4 serves as a temperature sensor for GABAergic WSNs in the POA of the hypothalamus, regulating body temperature and preventing hyperthermia. Manipulation of the Gαq-phospholipase Cβ (PLCβ) pathway can reduce the temperature sensitivity of WSNs, thereby partially inhibiting the effects of brain warming-induced hypothermia (Montell, 2012; Kamm et al., 2021; Zhou et al., 2023). This pathway has been demonstrated to mediate temperature sensitivity in Drosophila (Kwon et al., 2008; Shen et al., 2011). The physiological thermoregulatory mechanisms of WSNs and their genetic characterization remain inadequately understood, and the thermoregulatory mechanisms in animal models, such as mice, may differ from those in humans (Zhang et al., 2023). Moreover, technical limitations hinder the real-time monitoring of temperature-sensitive neuronal activity, highlighting the pressing need for the development of more precise techniques (Hori and Katafuchi, 1998; Vriens et al., 2014).

To address these challenges, we propose improvement strategies and future research directions:

Mechanistic level: Conduct a cell-type analysis of the connectomics of the POA to clarify the synaptic connectivity patterns between the ventral somatic neurons WSNs and other neurons, precisely locating the core neural circuits for thermoregulation. Do a multi-omics analysis of thermosensitive neuronal subtypes under thermal stress. Integrate transcriptomic, proteomic and metabolomic data to reveal temperature effects on key ion channel function and expression (e.g., TRP family), neuronal thermal signaling pathways and WSNs gene expression changes induced by heat stimulation. Real-time monitoring techniques and machine learning algorithms were introduced to optimize experimental methodologies, enabling the precise capture of WSNs activity patterns. This approach successfully overcame the technical barriers associated with real-time tracking of temperature-sensitive neurons.

Translational level: The drugability of potential molecular targets TRPM2/TRPC4 must be validated, and their specificity and safety as thermoregulatory targets thoroughly investigated. The intervention window must be defined. BBB-penetrating tool compounds must be developed, and targeted drugs designed using nanoengineering techniques to overcome central delivery barriers and enhance the targeted regulation efficiency of WSNs. Cross-species research must be conducted. Use multi-omics technologies to analyse physiological responses and differences in gene expression across species during heat exposure. This will help to establish more precise animal models and complete pharmacokinetic/pharmacodynamic (PK/PD) studies in large animals. This will bridge the mechanistic gap between small animal models and humans (Zhang et al., 2023); Explore the translational applications of optogenetics and non-invasive neural modulation technologies. These technologies have achieved breakthroughs in developing solutions to restore vision and in treating epilepsy and depression (Fakhoury, 2021; Lin et al., 2022; Gao et al., 2023; Ledri et al., 2023; Yan B. et al., 2023). These approaches allow for the precise control of neural circuits associated with heatstroke, providing the technical foundation for targeted interventions.

Clinical level: Carry out practical trials to test the effectiveness of “accelerated cooling combined with drug or neuromodulation therapy.” This means using targeted drugs with neuromodulation technologies to improve the effectiveness of existing physical cooling methods and improve patient outcomes. Identify the neural circuits and neuronal types relevant to heatstroke treatment in order to design personalized therapeutic strategies based on patient variability and thereby expand treatment options. Advance the clinical translation of non-invasive thermoregulation technologies. Combine physical cooling with precision intervention using targeted drug delivery systems to create a new treatment model, making it more feasible for patients and easier to use.

In conclusion, WSNs are significant in thermoregulation. However, their functions and thermoregulatory mechanisms necessitate further investigation. It is anticipated that further research will elucidate the functions and of WSNs, establish a scientific foundation for clinical applications, identify novel molecular targets for temperature regulation, and enhance treatment efficacy for patients experiencing heatstroke. Cross-species comparative studies will enhance the understanding of thermoregulation differences and similarities among various animals, offering valuable insights into the prevention and treatment of heatstroke.
